# A Population Health Approach to Address the Burden of Congenital Heart Disease in Kerala, India

**DOI:** 10.5334/gh.1034

**Published:** 2021-10-18

**Authors:** Sreehari M. Nair, Bistra Zheleva, Adriana Dobrzycka, Peter Hesslein, Rajeev Sadanandan, R. Krishna Kumar

**Affiliations:** 1 National Health Mission, Kerala, Thiruvananthapuram, IN; 2 Children’s HeartLink, Minneapolis, Minnesota, US; 3 Health Systems Transformation Platform, Delhi, IN; 4 Department of Pediatric Cardiology, Amrita Institute of Medical Sciences, Amrita Viswa Vidyapeetham, Kochi, IN

**Keywords:** Congenital heart disease, Congenital Heart Surgery, Population health, Newborn Screening

## Abstract

**Background:**

Congenital heart disease (CHD) has emerged as a leading contributor to infant mortality in many low-and middle-income countries (LMICs). We report early results of a population health program for CHD, implemented in the state of Kerala, India.

**Objective:**

Report on early results of a population-based program implementation in a LMIC to reduce mortality from CHD.

**Methods:**

We retrospectively analyzed the results of an innovative population-based program to address CHD. We devised, implemented and evaluated measures in the care continuum to address deficiencies in CHD care in Kerala, India, through structured capacity building initiatives that enabled early detection, prompt stabilization and expedited referral to a tertiary center. A comprehensive web-based application enabled real-time case registration, prioritization of treatment referrals, and tracking every child registered with CHD. Advanced pediatric heart care was delivered through a public-private partnership.

**Results:**

Early identification, referral, and treatment of infants with CHD were improved. The web-based application, ‘*Hridyam*,’ registered 502 cases in 2017 (Aug–Dec), 2190 in 2018 and 3259 in 2019; infants < 1 year of age constituted 56, 62 and 63% in these years, respectively. The number of heart operations managed through *Hridyam* rose from 208 to 624 and 1227 in the same years, with overall 30-day mortality of 2.4%. Overall- and CHD-related infant mortality in Kerala fell by 21.1% and 41.0%, respectively, over the same interval. Unmet challenges include lack of universal catchment and a 5% preoperative mortality rate.

**Conclusion:**

We demonstrate successful implementation of a population-based and real-time approach to reduce CHD mortality. We speculate that *Hridyam* has contributed to the observed decline in Kerala’s IMR from 12 to 7 between 2016 and 2019. This model has potential applications for other conditions, and in other jurisdictions, especially LMICs considering building CHD capacity.

## Introduction

With the adoption of the Sustainable Development Goals in 2015, the United Nations set ambitious targets for the worldwide reduction of childhood mortality [1]. Many regions already had seen significant epidemiologic shifts in recent decades. From 1990 to 2018, the global infant mortality rate (IMR) had fallen from 65 to 29 deaths per 1,000 live births, largely through population-based approaches to communicable diseases and nutritional deficiencies [2].

A recent study [3] demonstrated that congenital heart disease (CHD) is the seventh contributor to global infant mortality, causing 180,624 infant deaths in 2017. But CHD assumes increasing importance in developing countries: the rank of CHD as a contributor to infant mortality increased between 1990 to 2017, from fourth to second in high-middle socio-demographic index countries and from fifth to fourth in middle socio-demographic index countries.

India represents an interesting microcosm of the global situation. With an overall IMR of 31/1,000 live births, there are regions of the country that continue to struggle with infectious diseases and malnutrition. Other regions more closely mirror the developed world. The Indian state of Kerala has had an IMR of 12 for more than a decade [4] but has set a goal of six per 1,000 live births by 2030 [5].

In 2012, concerned that its IMR had been stagnant for so long, the Government of Kerala commissioned the Indian Academy of Pediatrics, Kerala Chapter to evaluate the causes of IMR in the state [6]. That study showed that infant deaths from infection and malnutrition had significantly declined, and that birth defects were the leading cause of infant mortality (30%) and that a significant reduction in IMR would require addressing this burden. Among these, CHD represents the world’s most common class of major birth defects, affecting one in 120 newborns. About one fourth of all CHDs are considered critical congenital heart disease (cCHD) [7], which require a lifesaving procedure in the first year of life.

In 2012, the Government of India started the *Rashtriya Bal Swasthya Karyakram* (RBSK) [8], a national child health initiative for screening and treatment of childhood diseases and disabilities, including CHD. This program, administered by the National Health Mission (within the Ministry of Health and Family Welfare), provides funding and technical assistance to individual States. With the addition of funds and commitment by the Government of Kerala, adequate financial resources were available for an innovative population health approach to address the burden of CHD in the state.

## Methods

**Figure 1:**
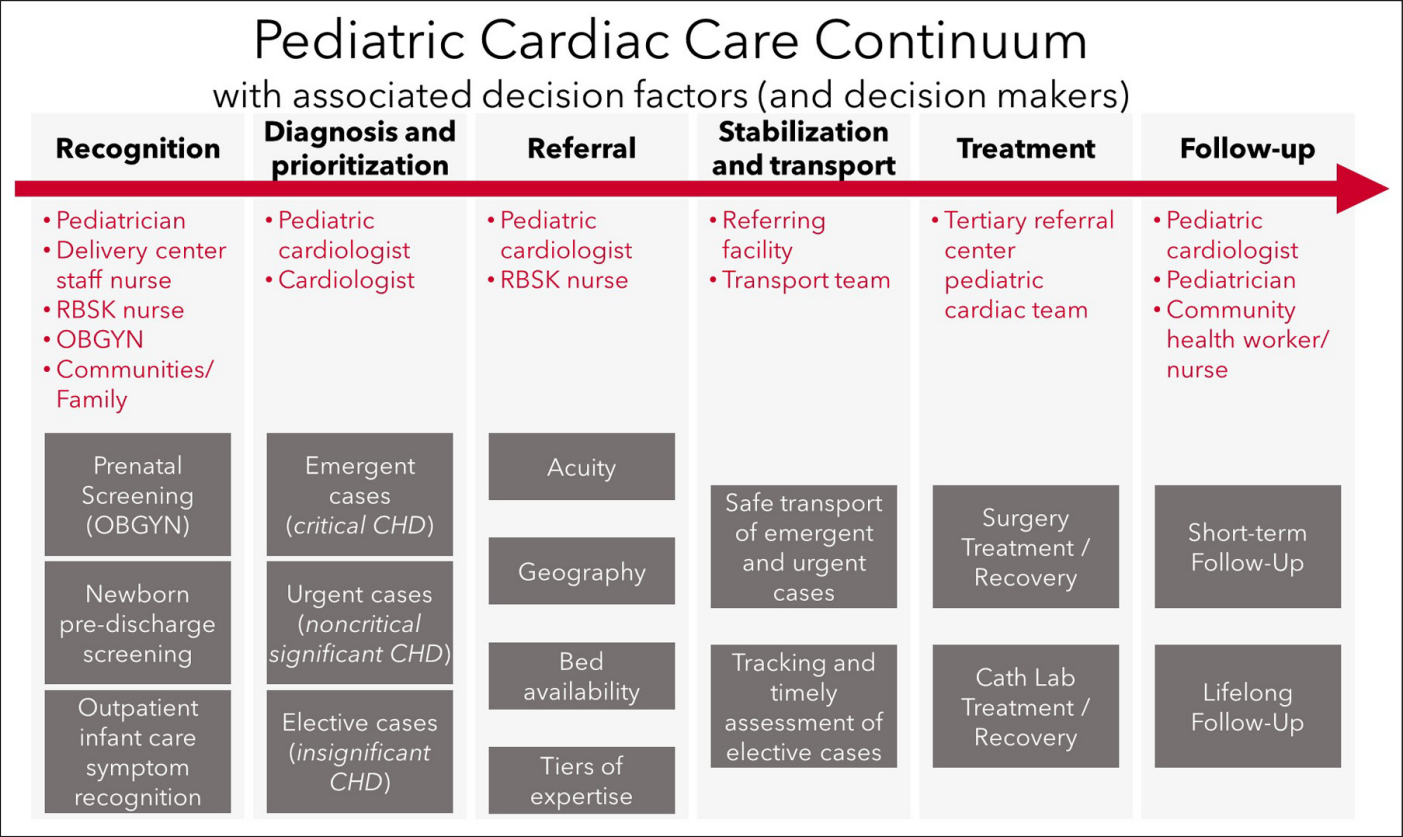
CHD Patient Care Continuum.

**Figure 2:**
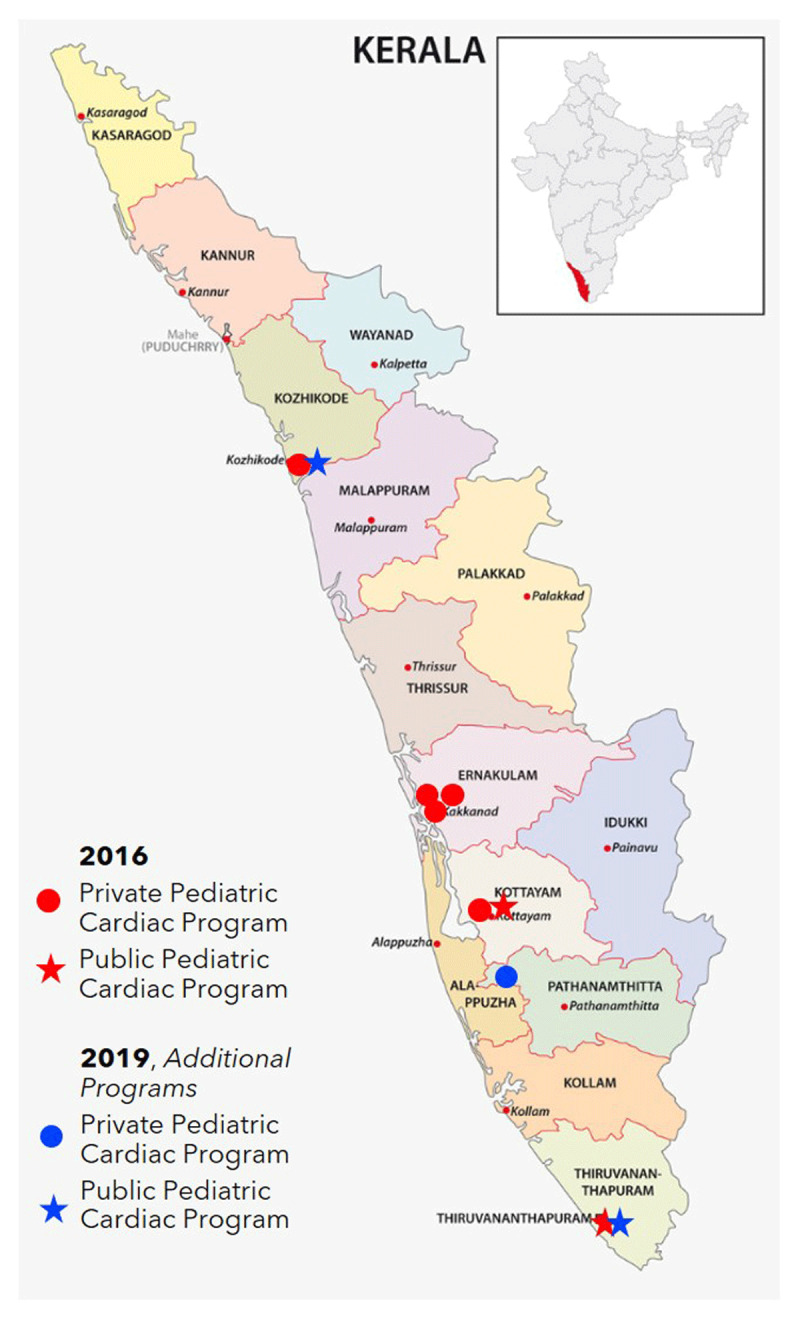
Kerala Pediatric Cardiac Care: Situation Analysis 2016 and 2019.* * Some private centers in the initial assessment were not empaneled (selected) by the government and later either discontinued their pediatric cardiac services or closed completely.

**Figure 3:**
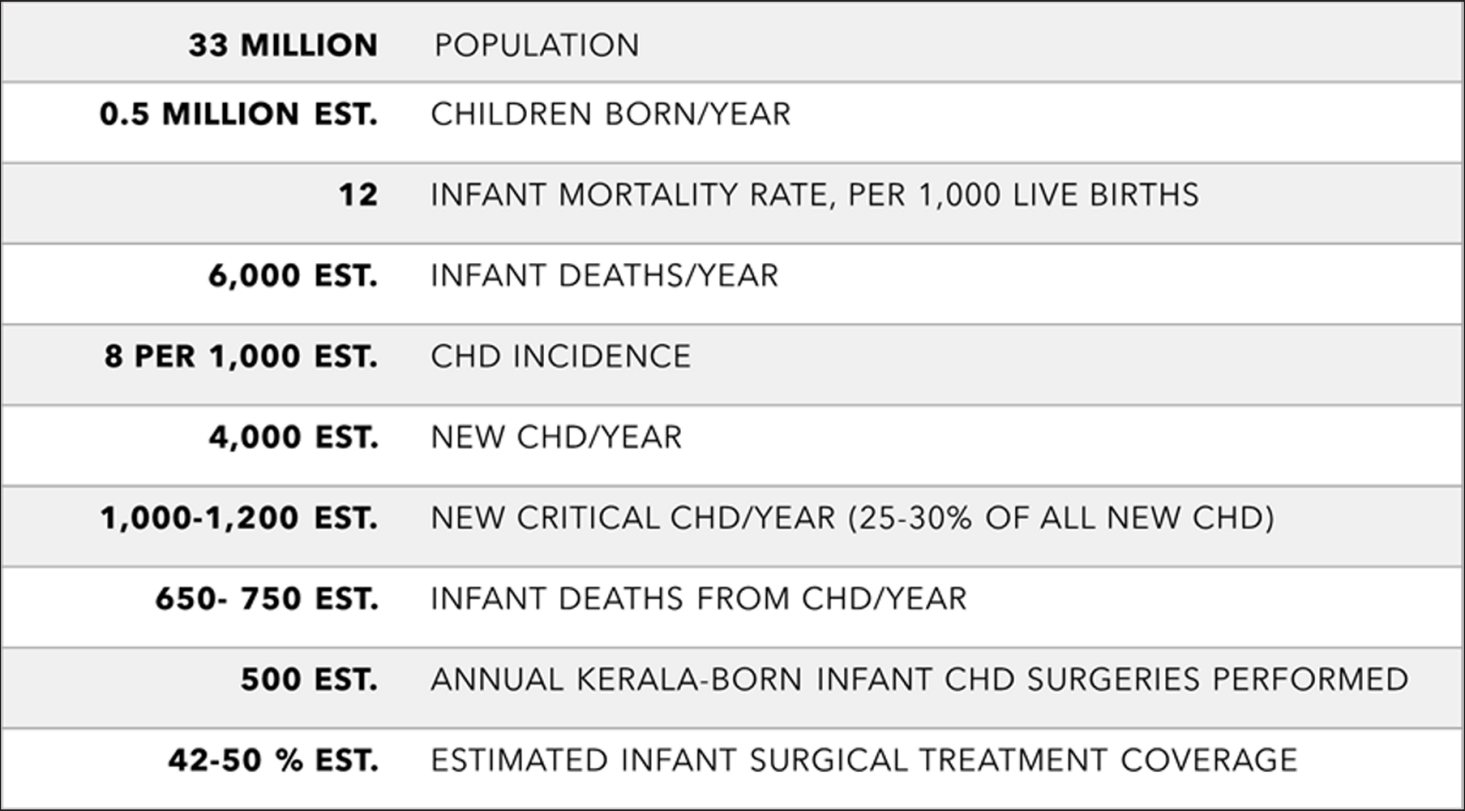
Congenital Heart Disease Demographics in Kerala, 2016. CHD: Congenital Heart Disease; est.: estimated.

**Figure 4:**
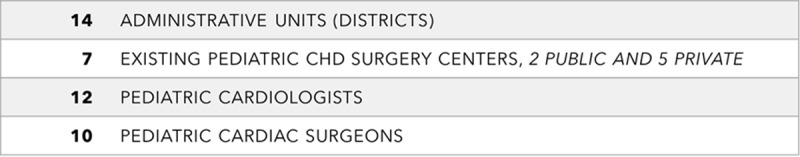
Kerala Capacity to Address CHD, 2016.

The Kerala government resolved to develop a comprehensive plan to address the problem of CHD in general and cCHD in particular. Consultations were held with various stakeholders representing the public and private health sectors; UNICEF Kerala; and Children’s HeartLink, a US-based nongovernmental organization (NGO). With input from stakeholders, Children’s HeartLink conducted an assessment and developed a continuum of care model (Figure 1) describing the lifetime path for such children [9], rather than viewing their care as a one-time surgical event. The care continuum is similar to a public health model described recently [10], but focuses more on development of clinical services, which reflects the gaps in most LMICs. Children’s HeartLink also conducted a survey of Kerala’s of existing capacities (Figures 2, 3 and 4), and assessed the systemic impediments to providing optimal cardiac care (Table 1).

**Table 1:** Challenges and Interventions within the Pediatric Cardiac Care Continuum in Kerala (see text for details).

	Challenges	Interventions Undertaken

**Recognition**	Limited awareness & expertise in fetal echocardiography Lack of newborn pulse oximetry screening & equipment Medical professionals inadequately trained to recognize CHD Little public concern for CHD	Obstetric ultrasound training Neonatal pulse oximetry program established Neonatal nurses perform pre-discharge physicals Pediatricians trained for early recognition Public awareness IEC campaign
**Diagnosis & Prioritization**	Spotty or nonexistent processes for assessment of suspected cases	*Hridyam* requires remote review & triage within 24 hours
**Referral**	Lack of an organized system to prioritize and refer patients to a treatment center causing dangerous delays	Immediate referrals, with diagnosis & geography considered
**Stabilization and Transport**	Limited understanding of how to stabilize sick infants with heart disease Absence of a neonatal transport network to get babies safely to a treatment center	Transport network developed by the government Web-based transport app now in pilot testing
**Advanced Pediatric Heart Tertiary Care**	Limited public-sector capacity to treat cCHD Limited access to private-sector capacity	Expansion of public-sector capacity at 3 institutions Collaboration with private sector in an effectively integrated system
**Follow up care**	No standardization or public health drivers of postoperative follow-up	Follow-up protocols developed and integrated within *Hridyam* Nursing group tasked with postop in-home follow-up visits

The continuum of care identified six stages of treatment, including the stage of surgical treatment, that would together contribute to a successful long-term outcome. Analysis of the challenges presented at each stage of the continuum led to a variety of interventions, also listed in Table 1 and a retrospective analysis of the outcomes is detailed in the Results section. The ultimate aim of this systems approach was to greatly increase the number of children receiving treatment and their chances of survival. The interventions were mostly implemented with technical assistance from Children’s HeartLink, and focused on improvement of the sensitivity and timeliness of cCHD recognition and referral, so that all such babies could make it to a treatment center in optimal preoperative condition, enhancing the surgical outcome which is then better-sustained by means of coordinated follow-up care.

**Figure 5:**
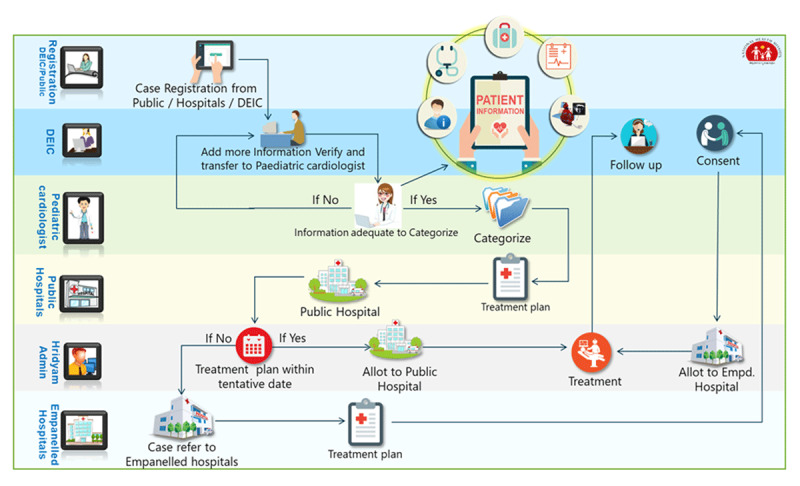
*Hridyam* Process Map.

In 2017, the Kerala government developed and launched a comprehensive web-based application, to accelerate each infant’s progression through the continuum stages, track their progress, and yield measurable outcomes (Figure 5). Named ‘*Hridyam*—for little hearts,’ it functions first as a registry for children 0–18 years with suspected CHD of all types. Any physician within Kerala can add a name; there are no layers of medical hierarchy or bureaucracy to impede or delay this process. Once registered, each child’s progress is coordinated at the local level by the District Early Intervention Center (DEIC, under the National Health Mission), but monitored centrally by the National Health Mission under Department of Health. The incentive for universal registration is that *Hridyam* serves as the sole entry point for accessing government-funded treatment via the above-mentioned government RBSK scheme. Once listed, a pediatric cardiologist is obliged to review the online record within 24 hours and classify the case according to urgency for treatment. (In situations of insufficient information, that same cardiologist may direct the DEIC to acquire further tests.) A referral is made, based on a protocol that favors geographically proximate public institutions, but liberally acknowledges when special public or private hospital expertise favors a more distant referral within Kerala. This act of triage and referral triggers a timeline for diagnostic fine-tuning and the development of a treatment plan. A new timeline then activates, and if treatment does not occur within an allotted interval, referral elsewhere is considered. Outcomes are tracked on the same website, and the *Hridyam* protocol also directs the nature and timing of follow-up care. Everything concerning each registrant’s case occurs in real-time, is tracked and directed as needed by the government agency and can be accessed by any interested and qualified in-state party.

Given that this report used aggregate population-level data from a government owned database, ethical/IRB approval and patient consent for participation were deemed unnecessary.

## Results

The *Hridyam* registry website went live in August 2017. Early results are reported below (Tables 2, 3 and 4). Steady growth in case registrations and surgical treatments is documented, as well as a trend toward newborn and infant procedures. Well before the activation of *Hridyam*, the Kerala government undertook a number of complementary initiatives to address the systemic deficiencies listed in Table 1. These interventions, categorized by their position in the CHD Care Continuum, are as follows:

**Table 2:** *Hridyam* Patient Registrations.

Year	Age 0–12 months	Age > 1 year	Total

2017 (Aug–Dec)	309	193	502
2018	1409	781	2190
2019	2237	1022	3259
**Total**	**3955**	**1996**	**5951**

**Table 3:** *Hridyam* Patient Surgical Profile.

Age	Year	Number of Cases (proportion %)	Most Frequent Procedures

**Under 1 month**	2017	17 (8.2)	
	2018	83 (13.3)	
	2019	166 (13.5)	
	**Total**	**266 (12.9)**	**Arterial switch, Patent Ductus Arteriosus (PDA) stent, Total Anomalous Pulmonary Venous Connection repair**
**1 month to1 year**	2017	99 (47.6)	
	2018	306 (49.0)	
	2019	614 (50.0)	
	**Total**	**1,019 (49.5)**	**Ventricular Septal Defect (VSD) repair, Tetralogy of Fallot repair, PDA device**
**Over1 year**	2017	92 (44.2)	
	2018	235 (37.7)	
	2019	447 (36.4)	
	**Total**	**774 (37.6)**	**Atrial Septal Defect (ASD) device, ASD repair, PDA device**

**Table 4:** *Hridyam* Surgical Outcomes.

Year	Surgical Cases	≤30-day Mortality (% of cases)	Late Mortality(% of cases)

2017 (Aug–Dec)	208	3 (1.4)	4 (1.9)
2018	624	22 (3.5)	21 (3.4)
2019	1,227	25 (2.0)	19 (1.5)
**Total**	**2,059**	**50 (2.4)**	**44 (2.1)**

### Recognition

1. *Prenatal screening and diagnosis*: Since early recognition is the key to saving lives in cCHD and obstetricians and sonographers are most likely to pick up the cases during the antenatal period, they were encouraged to actively search for CHDs. Seven training sessions were held, to enhance the prenatal diagnostic skills of 603 obstetrical and radiological sonographers. The Kerala Ministry of Health and Children’s HeartLink conducted a 6-month follow-up evaluation of fetal echo training participants. Of 185 public sector participants who were contacted, 50 responded. Ninty-six percent reported that the workshop improved their skill in screening and identification of CHD. The majority of participants reported feeling comfortable capturing the essential three-vessel (84%) and the outflow tract (76%) views. All participants reported increasing the number of fetal heart screening procedures three months after the training as compared to the three months prior. More than 200 fetal cases of CHD have been registered in the *Hridyam* portal.

2. *Newborn physical examination*: A systematic protocol for pre-discharge newborn physical exams has been developed at all public hospital birth centers, and a cadre of 196 government nurses is being trained for its implementation.

3. *Neonatal pulse oximetry screening*: A neonatal pulse oximetry screening program was established at all government delivery centers. Many studies have shown that detection of CHD in newborns is greatly enhanced by the addition of pulse oximetry to the newborn physical exam [11,12]. Ninety-eight health centers received pulse oximetry devices, and three rounds of trainings were completed at three different levels of the health system to establish a neonatal pulse oximetry screening program at all government delivery centers. In all, 371 nurses have been trained to execute this program. Through 2019, this program enabled the screening of 157,295 (76.8%), out of a potential 204,883 newborns. Pulse oximetry has been responsible for identifying 134 cases of CHD, including 74 critical cases.

4. *Post-natal screening*: Not all CHD is apparent at birth. Even some critical conditions may not present until after hospital discharge. To capture these cases, pediatricians were trained to improve their diagnostic skills. Four full-day workshops were held where 262 pediatricians received training on the presenting signs and symptoms of CHD. In 2019 a weekly online series of lectures focused on pediatric heart disease was initiated. So far, 51 sessions have been held with an average participation of 120 clinicians.

5. *Publicity*: In order to create awareness and generate demand, caregivers and the general public were informed of the *Hridyam* program and its goals through a statewide community health education and promotion campaign.

### Diagnosis, Prioritization and Referral

1. To establish a precise diagnosis and to trigger appropriate action, *Hridyam* requires record review and patient triage by a designated panel of pediatric cardiologists within 24 hours of a child’s registration into the system.

2. To facilitate communication with patients’ families, text messaging was added to *Hridyam* to notify parents of consultation appointments, surgical dates, and other important patient-related information.

3. A primary aim of the *Hridyam* system is to avoid delays in referral. Treatment centers have reported that babies with cCHD are now arriving sooner, and in better condition, than in the past. We hope to document this observation in a subsequent report.

### Stabilization and Transport

1. A transport network has been established in Kerala.

2. Destabilization of sick babies during transport can significantly add to the morbidity and costs of overall care, and the inability to monitor during this critical phase of the care continuum constitutes a hazardous blind-spot. One of the *Hridyam*-participating hospitals has developed and implemented an mHealth innovation called NeoPORT, a smartphone-based communication system that connects the three major stakeholders of any newborn transport (sender, transporter and receiver) on a common data-sharing and real-time-monitoring platform [13]. The system integrates checklists for each team, seamless real-time communication of vital clinical data, GPS tracking, and clinical scoring systems to enhance vigilance, and safety.

### Treatment

1. Definitive surgery remains the *sine qua non* event around which all cCHD treatment revolves. As of 2016, seven surgical centers had been identified in Kerala, with the capacity to treat 42–50% of the estimated new cases of cCHD annually (Figure 2). The Government of Kerala since then took steps to address the deficit by developing one new public hospital center, augmenting one existing public center, and empaneling one more private hospital. One more government hospital center is slated for development.

2. Table 4 documents the nature and number of treatments effected under the *Hridyam* program. Although designed to encompass all CHD, *Hridyam* includes systems nimble enough to recognize and respond to the acuity of cCHD. This is reflected in the timing and nature of the procedures performed, as well as the trend toward younger age at operation. In 2019, 63.5% of *Hridyam* operations were accomplished during infancy (Table 3).

### Follow-up

1. A post-procedure follow up protocol was developed, and is actively monitored for compliance, within the *Hridyam* system. This mechanism also enables the government to track patients longitudinally.

2. A cadre of 1,040 government nurses who operate through the local DEIC in Kerala to fulfill the broad mission of RBSK, have been trained and tasked to perform a schedule of in-home pre- and post-operative follow up examinations of CHD patients, and to report them on the *Hridyam* portal.

### Reduced Mortality

**Figure 6:**
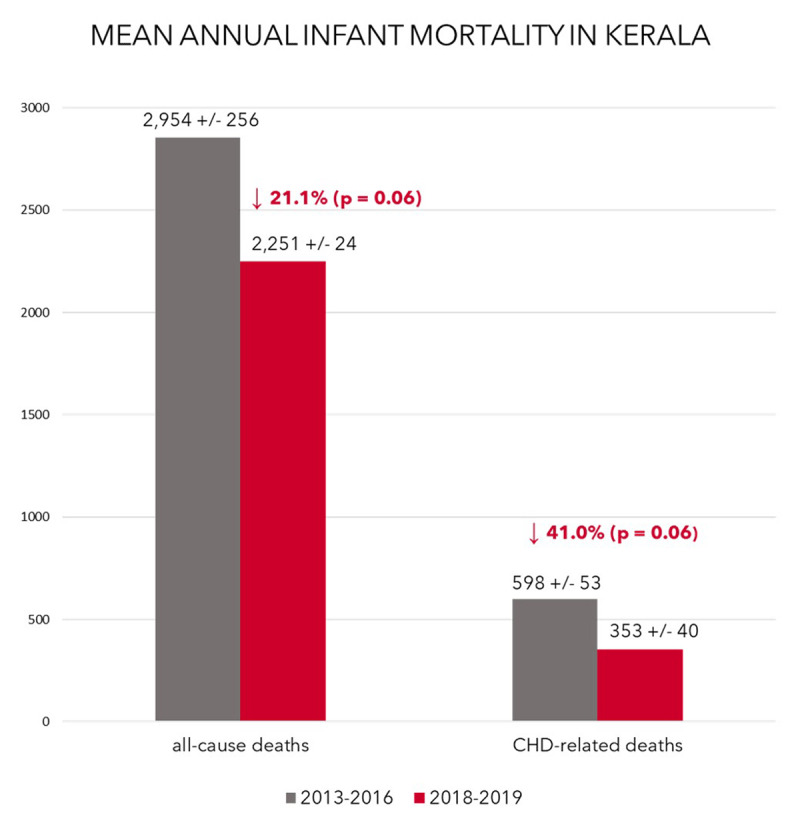
Reduction in all-cause and CHD-related infant mortality following the introduction of *Hridyam* programs in Kerala. By the non-parametric Mann Whitney U test, this improvement is of borderline statistical significance (p = 0.06), probably due to the small sample size of comparing data from two *Hridyam* years to four pre-*Hridyam* years.

Figure 6 compares total- and CHD-related infant mortality in Kerala, before and after undertaking this population-based program. The average annual number of all-cause infant deaths for the four years before *Hridyam* implementation (2013–2016) was 2,954 +/– 256. Following establishment of this program (2018–2019) this number fell to 2,251 +/– 24, a decline of 21.1 percent (p = 0.06). Comparing the same time intervals, the average annual number of infant deaths attributed to CHD fell even more substantially, from 598 +/– 53 to 353 +/– 40, a reduction of 41.0 percent (p = 0.06).

## Discussion

The medical literature regarding CHD reflects an impressive evolution over the past eight decades, from the autopsy table to the examination table to the operating table and beyond. But reports have generally been piecemeal, documenting medical treatment advances for specific conditions rather than utilizing a public health/population health perspective. Against an enormous backdrop of communicable diseases and nutritional deficiencies, it has been difficult to view congenital cardiac defects as a significant public health concern.

The last two decades have been characterized by rapid and substantial improvements in human development indices. As the Global Burden of Disease study revealed, CHD has emerged as an important cause of remaining mortality among newborns and infants, even in LMICs [3]. However, CHD treatment requires a complex and multidisciplinary team approach, which most LMICs are ill-equipped to provide. In most LMICs, pediatric heart care is currently delivered in a select few tertiary centers that can only cater to a small fraction of affected children. Moreover, the pre-hospital aspects of the CHD care continuum receive little attention, further limiting the number of children presenting for advanced treatment and burdening the care centers with patients in deteriorated condition.

These same recent decades have also witnessed exponential growth in our ability to collect and act upon health data in real time. We present our experience implementing a comprehensive population-based program, specifically aimed at reducing the mortality and morbidity from CHD by targeting all the elements of the care continuum in the state of Kerala.

Our retrospective analysis of the start of the *Hridyam* program in Kerala shows that it is among the first to use technology in order to optimize timely management of CHD patients, according to need and case urgency, by creating a statewide network that connects primary health centers to tertiary hospitals. Another strength of the *Hridyam* program is its focus on all elements of the care continuum, from prenatal and newborn diagnosis to surgical post-hospital follow-up care. We believe the initial results justify optimism for the future of this approach. Between 2016 and 2018, Kerala reported a decline of its IMR from 12 to 7 per 1,000 births and newborn deaths attributed to CHD fell 41.0% [14]. This improvement is of borderline statistical significance (p = 0.06), probably due to the small sample size of comparing data from two *Hridyam* years to four pre-*Hridyam* years. While this association does not prove a relationship, we hope that further studies will confirm that the *Hridyam* program has contributed substantially to this success. This program sprang from a fortunate alignment of several factors: national and Kerala goals for reducing infant mortality to a single digit; data from state-commissioned study revealing that the decline has to come from addressing congenital anomalies (of which CHD has the largest share); government partnership with private hospitals to provide technical guidance; NGO partner with the ability to provide technical assistance and additional funding; and national level financing through the RBSK. *Hridyam* was further driven by population data to make a compelling case to policymakers, by political willingness and commitment of government champions to make an investment.

*Hridyam* is a government-run program, created to address a data determined goal, but it could not have succeeded without the government’s willingness to assess the existing capacity in and partner with private providers for these highly specialized services, understanding the deficiencies and realizing that building them *de novo* takes time and lots of resources. Similarly, the private sector’s corresponding willingness to cooperate was key to the successful implementation. This collaboration extends well beyond the care of specific cases: public and private clinicians are key participants in the educational and triage aspects of *Hridyam* [15]. For its part, the government has provided most of the funding, software development, data management, and the development of nursing and transport networks. Strong and transparent involvement by an NGO has facilitated the partnership and provided access to learning from international experts.

The *Hridyam* program continues to undergo constant reassessment and modification, as new data emerge. Although *Hridyam* has been developed and financed within existing budgets of several government agencies and stakeholders, its overall cost has not been fully assessed. It bears mention that the costs to develop and administer *Hridyam* have been negligible compared to the costs of patient care, and we hope to demonstrate that per-patient costs may in fact be reduced by the quicker and healthier referral patterns attributable to *Hridyam*. For this program to be sustainable in the future, it must remain attentive to the support and training of caregivers at all levels. Additionally, the program should continue to assess the treatment capacity in the state in both volume and complexity. Ongoing efforts to improve early CHD detection and infant transport will be needed. Ultimately, the program will require continued commitment and funding support from succeeding state and central governments.

We believe that *Hridyam* has had beneficial effects on infant mortality in Kerala, and on the overall course for children in Kerala with CHD. In the meantime, we believe that such a population-based, integrated and real-time approach has potential application well beyond CHD, to other public health concerns and within a broad range of jurisdictions, especially in LMICs.

## Conclusions

To our knowledge, there are no examples of implementation of a comprehensive population health program addressing congenital heart disease in a low- and middle-income country. The UN Sustainable Development Goals target for reduction in infant mortality and the increased contribution of CHD to infant mortality provide an opportunity to make a compelling case to policymakers for investment in the development of pediatric cardiac services across the care continuum.

We believe this is the first report of a successful implementation of a population-based approach in a low and middle-income country to reduce mortality from congenital heart disease through the implementation of a capacity building activities focused on all elements of the care continuum, from prenatal and newborn diagnosis to surgical post-hospital discharge follow-up care. This report describes the necessary prior health system capacity assessment as a prerequisite for a successful implementation and also highlights how a public-private collaboration led to improved access and lower infant mortality.

We speculate that this program has significantly contributed to the observed decline in Kerala’s infant mortality rate from 12 to 7 per 1,000, and the 41.0% fall in infant CHD deaths, between 2016 and 2019. As countries set goals for reduction of infant mortality and realize the gains from addressing the burden of infectious diseases and malnutrition, the share of mortality from CHD will increase and LMICs are likely to face shortage of capacity and resources. This study provides evidence for one avenue to achieve reductions regionally in such settings. The model we present has potential applications for other conditions, and in other jurisdictions.

### Limitations

Our experience has revealed that *Hridyam* has some shortcomings. While the system is fully integrated and comprehensive, its screening programs are compulsory only in public birth centers, accounting for about 30% of statewide deliveries (at the time of the initiation of *Hridyam*, the state government lacked the power to regulate private birth centres) [16]. Preoperative mortality among registered cases remains high (around 5%), and although it is trending downward, most of these babies had conditions for which surgery is not being offered (hypoplastic left heart syndrome and complex single ventricle). Although cCHDs were picked up through newborn screening, the confirmation of cases remains a limiting factor due to the insufficient echocardiography services in the state, a frequent challenge in LMICs. *Hridyam*’s triage and treatment arms do not capture all cases, because of the option for patients to forgo government funding by charting their own course to private hospitals. Some aspects of the *Hridyam* program are unfunded or underfunded, such as triage evaluations and postoperative follow-up visits, and *Hridyam* has further revealed that different institutions have different cost structures, making integration under the RBSK scheme challenging.

Finally, this program owes much of its success to the innovative online registry *Hridyam*, which allows instant registration, algorithmic referral, and real-time tracking throughout the care continuum. Success is also driven by strong drive form policy makers, a public funding mechanism, and by enthusiastic collaboration between public and private treatment centers. While we believe this is a model to be explored in countries at all levels of socio-economic development, we acknowledge that, such a program relies on a level of infrastructure that may not be available in every low-to-middle-income region.

## Data Accessibility Statement

Data of this report will be available upon request.
